# Surgical results with the use of Silicated Calcium Phosphate (SiCaP) as bone graft substitute in Posterior Spinal Fusion (PSF) for Adolescent Idiopathic Scoliosis (AIS)

**DOI:** 10.1186/s13013-015-0051-x

**Published:** 2015-08-21

**Authors:** Nanjundappa S. Harshavardhana, Mohammed H H Noordeen

**Affiliations:** Twin Cities Spine Center, 1111 S 8th St, Apt 214 N, Minneapolis, 55404 MN USA; Spinal Deformity Unit, Royal National Orthopaedic Hospital NHS Trust, Brockley Hill-Stanmore, Middlesex HA7 4LP UK

**Keywords:** *Silicated Calcium Phosphate (SiCaP)*, *Idiopathic Scoliosis (IS)*, *Posterior Spinal Fusion (PSF)*, *Bone Graft substitutes*

## Abstract

**Background:**

The *gold standard* iliac crest bone graft (ICBG) used to achieve arthrodesis in spinal fusions is not without complications (donor-site morbidity, iliac wing fractures etc.…). Our objectives were to evaluate the role of silicated calcium phosphate (SiCaP), an osteoconductive synthetic bone graft substitute in conjunction with locally harvested autologous bone in achieving arthrodesis following posterior spinal fusion (PSF) for adolescent idiopathic scoliosis (AIS) and report clinic-radiological results / adverse events with its use in a prospective single surgeon case series (Level of evidence [LoE] IV) treated by low implant density index (IDI) constructs (i.e., IDI ≤1.5).

**Methods:**

Thirty-five patients (8♂ & 2727♀) who underwent PSF and followed-up for a minimum of 2 years formed the study cohort. The mean age at surgery was 15 years (range: 11–21y) and pre-op Cobb angle was 60° (range: 40°–90°). SiCaP mixed with locally harvested bone during exposure and instrumentation was laid over instrumented segments. The average SiCaP used per patient was 32mls (range: 10–60mls). Radiographs were assessed for fusion at serial six monthly follow-ups. All clinical adverse events and complications were recorded.

**Results:**

The mean follow-up was 2.94 years (range: 2–4y). The post-op Cobb angle improved to 23° (range: 2°– 55°) and the mean in-patient stay was 7.72 days (range: 5–13d). The mean number of instrumented segments was 9.4 (range: 4–13) and implant density index (IDI) averaged 1.23 (range: 1.15–1.5). Radiographic new bone formation was seen within 3 months in all cases. All patients (except two) were highly satisfied at minimum follow-up of 8 years. There were two complications warranting revision surgery (deep infection, and implant failure without any evidence of pseudarthrosis). There were no SiCaP specific adverse events in any of the 35 patients.

**Conclusion:**

SiCaP facilitated early bony consolidation in operated cohort of AIS patients treated by PSF. There were no inflammatory reaction or other adverse effects associated with its use. SiCaP is a safe alternative to autologous iliac crest bone graft with reduced complications, morbidity, faster recovery and similar infection/fusion rates reported in the literature.

## Background

Adolescent Idiopathic scoliosis (AIS) is characterized by a frontal plane deformity of the spine and its exact etiology is unknown. Numerous theories are proposed to explain its etiology and pathogenesis [1]. Cobb angle is used to quantify and assess progression of deformity on standard PA radiographs, which have a three-fold reduced radiation to breast tissue than AP x-rays [2, 3]. Surgery is recommended and performed when the Cobb angle is ≥50°. Surgery addresses the shoulder, flank asymmetry and arrests progression of deformity. It also enhances self-esteem, improving body self-image, overall appearance and quality of life [4].

The first reported use of instrumentation for scoliosis was by Harrington [5]. Emphasis on achieving sound arthrodesis is attributed to JH Moe who recommended facetal and inter-laminar fusion [6]. Autologous iliac crest bone graft (ICBG) is considered to be gold standard in achieving arthrodesis for idiopathic scoliosis [7]. Numerous studies have reported excessive blood loss, iliac wing fractures, limited bone stock, prolonged hospital stay, increased pain and donor site morbidity with harvest and use of ICBG [8, 9]. Allografts and other synthetic bone graft substitutes are commonly used to minimise the risks associated with use of ICBG. The biological properties of allografts are variable and greatly influenced by processing techniques employed. Price reported 28 % pseudarthrosis rates with use of allografts for spinal fusions in comparison to 12 % for ICBG [10].

Silicated calcium phosphate (SiCaP) is a synthetic and porous bone graft substitute (ACTIFUSE™; ApaTech and Baxter Ltd, Elstree, Hertfordshire; UK) that is manufactured against highly controlled specifications mimicking the trabecular architecture of native cancellous bone as granules. It is available for use in two sizes with granule diameters of 1–2 and 2–5 mm. The silicate substitute at 0.8 % concentration by its negative surface charge significantly improves bone formation by increasing vascularity to host bone [11]. Studies have also demonstrated accelerated bone formation, enhanced volume of new bone formed and consolidation with remodelling into mature bone in-vivo animal studies [12]. SicaP’s use is well-documented and reported for degenerative spinal disorders. No published literature exists till date reporting its use in isolation (i.e., without mixing with vertebral or bone marrow aspirate or ICBG) in scoliosis surgeries and long spinal fusions. The purpose of this prospective clinical study was to evaluate the clinical and radiographic outcomes with its isolated as a bone graft substitute in conjunction with locally harvested bone in AIS patients treated by posterior spinal fusion (PSF) with low implant density index (IDI) constructs.

## Methods

Thirty five consecutive patients with AIS scheduled for posterior spinal fusion at our institution were enrolled into this prospective study against pre-determined stringent inclusion criteria following a research and ethics committee (REC) approval which were:

i. Age at surgery – at least 11 years
ii. Cobb angle of structural thoracic curve ≥50° and that of thoraco-lumbar/lumbar curve ≥40°.
iii. Patients/parents & care givers signed a written consent form voluntarily agreeing to participate in the study.

The exclusion criteria included:

i. AIS treated by anterior or combined anterior + posterior surgery
ii. Revision spinal surgeries and scoliosis secondary to other etiologies (i.e., congenital/neuromuscular/syndromic etc.…)
iii. Patients with known malignancy or local/systemic infection
iv. Those who refused to provide consent to participate in the study.

**Fig. 1:**
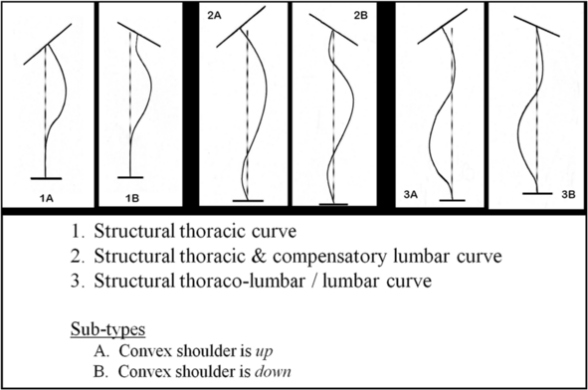
The Noordeen AIS curve algorithm: Six curve patterns

Information brochure about ACTIFUSE™ was provided to enrolled patients and all questions were convincingly answered by the surgical team before their surgeries. The senior author (MHHN) has devised a surgical algorithm that identifies all AIS curves as three main curve types (I-III). Each curve type is further sub-divided into sub-type A & B depending on whether the convex shoulder was higher or lower to inter-clavicular line thus yielding six curve patterns that all AIS curves fitted into. The definition of these three curve types with illustrative line diagram is illustrated in Fig 1.

### Surgical procedure

All patients underwent scoliosis correction by third generation instrumentation through a midline posterior approach by means of a segmental all pedicle screws or hybrid construct plus cobalt-chrome rod system. Three instrumentation systems used were:-

- Expedium Spine System (De-Puy Spine Inc., Raynham, Massachusetts, USA)
- Synergy Spine Instrumentation (Interpore Cross International, Irvine, California, USA).
- K2M Spine Solutions (K2M Inc., Leesburg, Virginia, USA)

After appropriate exposure of the spine by meticulous subperiosteaal dissection and haemostasis, facetectomies were performed. Cell saver and transfusion recycler was used in all cases and intra-operative estimated blood loss (EBL) was recorded as percentage of estimated blood volume (EBV). Pedicle screws were instrumented in the selected vertebrae after confirming the upper and lower instrumented vertebrae (i.e., UIV and LIV) with intra-operative fluoroscopy. Alternating convex and concave pedicle screws were inserted into the vertebrae (i.e., each vertebra had only one pedicle screw) except the end vertebrae, which had bilateral pedicle screws in all 35 patients. The implant density index (IDI) was no more than 1.5 in all cases. A pre-bent and appropriately contoured rod in sagittal plane was then inserted and correction was accomplished by a combination of the cantilever and translation manoeuvres. The convex (right sided rod) was inserted first to aid in translation and application of cantilever forces for correction. Finally, direct vertebral rotation was performed to correct the axial deformity. At the end of surgical correction, the spinous processes were resected and outer cortices of laminae were decorticated. 20–60mls of ACTIFUSE™ (depending on the no. of vertebral segments to be fused and quantity of locally harvested bone) was mixed at 60:40 ratio (i.e., 60 % of SiCaP and 40 % of autograft). The resulting composite material was laid over the prepared bony bed across the entire instrumented segment of the spine prior to the closure to facilitate arthrodesis.

### Postoperative care and rehabilitation

Swimming, Pilates and gentle stretching was encouraged at 6 weeks onwards. Cycling and non-contact sports permitted at 6 months postoperatively. Contact sports and horse riding was prohibited for up to a year. None of the patient wore any orthosis in the postoperative period.

### Radiographic & statistical analysis

All patients had preoperative standardized long-cassette erect PA and lateral radiographs. Supine side- bending views were performed to assess flexibility of structural and compensatory curves to aid decision-making in performing a selective spinal fusion. The senior author has devised a surgical algorithm and classified AIS into three curve main types. This simplified surgical algorithm also aided us in choosing fusion levels and was strictly adhered to for all cases.

The curve characteristics of all patients as per the senior author’s algorithm is summarised in Table 1. Serial post-op radiographs were performed at the time of discharge, 3, 6, 12 and 24 months post-op follow-up. The radiographs were evaluated for instrumentation failure, lucency around the screws, anchor pull-out or dislodgement from its initially inserted position and for defects in the fusion mass as indicators of pseudarthrosis by a fellowship trained junior colleague with input from an independent Consultant radiologist who was not involved in the patients’ treatment. Cobb angles of structural and compensatory curves were measured pre and serial postoperative radiographs using the same vertebral segments as preoperative x-rays. The degree of postoperative curve correction and loss of correction with time at final follow-up was diligently recorded. Pseudarthrosis when present was recorded as definite or probable. A definite non-union existed when proven by a CT scan (on 1–2 mm fine-cut osteo window) and presence of frank defect during revision surgery Pseudoarthrosis was considered as probable when:

**Table 1:** Results - Summary of *perioperative parameters* in all 35 patients

Patient ID	Sex	Age at Sx	F/u in mo	Levels fused	Instrumented Segments	IDI	Actifuse wt. in mls	Total Blood loss (EBV)	Intra-op BT^a^	Post-op BT^a^	Pre-op VAS	VAS at Discharge	Final f/u VAS	Complication	Fusion	Instrumentation system
1001	F	21	48	T2-L1	11	1.18	40	0.2	0	0	2	1	1	None	Good	Synergy
1002	F	19	30	T3-L3	12	1.16	20	0.3	0	0	2	1	0	None	Good	Synergy
1003	M	19	30	T2-L2	12	1.16	40	0.4	0	0	1	0	0	None	Good	K2M
1004	F	14	30	T3-L1	10	1.2	20	0.25	0	0	1	0	0	None	Good	K2M
1005	F	14	48	T4-L2	10	1.2	20	0.35	0	0	0	0	0	None	Good	Synergy adult
1006	F	16	48	T3-L1	10	1.2	40	0.3	0	0	0	0	0	None	Good	K2M
1007	M	14	48	T3-L3	12	1.16	40	0.45	0	0	0	2	0	MRSA deep infection	Good	Synergy
1008	F	14	24	T3-L3	12	1.16	40	0.35	0	0	0	0	0	None	Good	Synergy
1009	F	12	48	T3-T11	8	1.25	40	0.25	0	0	2	1	0	None	Good	Synergy
1010	F	14	48	T3-T11	8	1.25	30	0.3	0	0	2	0	0	None	Good	Synergy
1011	F	13	36	T4-T10	6	1.33	20	0.2	0	0	2	0	0	None	Good	Synergy
1012	F	13	30	T2-T12	10	1.2	25	0.25	0	0	3	2	0	None	Good	K2M
1013	M	15	48	T3-L3	12	1.16	40	0.3	0	0	0	0	0	None	Good	K2M
1014	F	11	30	T2-T12	10	1.2	40	0.3	0	0	0	0	0	None	Good	K2M
1015	F	13	24	T3-L1	10	1.2	40	0.3	0	0	0	0	0	None	Good	K2M
1016	F	13	30	T2-L1	11	1.18	30	0.3	0	0	6	2	1	None	Good	Synergy adult
1017	M	15	30	T3-T11	8	1.25	30	0.25	0	0	2	1	0	None	Good	K2M
1018	F	14	48	T4-T12	8	1.25	30	0.25	0	0	3	1	1	None	Good	Synergy
1019	F	19	48	T2-L3	13	1.15	40	0.35	0	0	0	0	0	Right rod at T9 is broken	Good	K2M
1020	F	15	48	T2-L2	12	1.16	60	0.35	0	0	0	0	0	None	Good	K2M
1021	F	18	24	T4-L2	10	1.2	35	0.28	0	0	0	0	0	None	Good	Synergy adult
1022	F	12	24	T4-L2	10	1.2	40	0.3	0	0	3	1	0	None	Good	K2M
1023	M	16	24	T3-T11	8	1.25	40	0.25	0	0	2	1	0	None	Good	K2M
1024	F	20	24	T10-L3	5	1.4	20	0.25	0	0	0	0	0	None	Good	K2M
1025	F	13	24	T2-L2	12	1.16	30	0.3	0	0	0	0	0	None	Good	K2M
1026	F	20	24	T2-T12	10	1.2	20	0.3	0	0	0	0	0	None	Good	K2M
1027	F	14	24	T3-T12	9	1.22	20	0.25	0	0	3	1	0	None	Good	K2M
1028	F	14	48	T10-S1	8	1.25	40	0.2	0	0	8	5	2	None	Good	Expedium
1029	M	17	36	T3-L3	12	1.16	40	0.3	0	0	3	3	0	None	Good	K2M
1030	M	14	30	T4-L1	9	1.22	30	0.25	0	0	4	4	2	Unhappy with pain relief	Good	K2M
1031	M	15	36	T3-T11	8	1.25	20	0.22	0	0	5	3	2	Had low back pain (NSAIDs)	Good	K2M
1032	F	16	48	T11-L3	4	1.5	25	0.25	0	0	1	0	0	None	Good	K2M
1033	F	14	42	T4-T11	7	1.28	30	0.35	0	0	0	0	0	None	Good	K2M
1034	F	13	24	T4-T10	6	1.33	30	0.3	0	0	3	0	0	None	Good	K2M
1035	F	12	30	T4-T10	6	1.5	20	0.25	0	0	2	1	0	None	Good	K2M

^a^ *BT* blood transfusion

- There was loss of correction by >10° in comparison with immediate postop x-ray
- Radiologically visible defect in the fusion mass
- Persistent axial back pain of intensity severe to warrant regular intake of pain-killers.

Thus collected clinico-radiological data was analysed using statistical package for the social sciences v16 (SPSS Inc. IBM Corp., U.S.A). Statistical significance was set at *p* < 0.05.

## Results

The study cohort comprised of 8 males and 27 females. The mean age at diagnosis of AIS was 10.75 years (range: 10 – 16). The mean age at surgery was 15 years (range: 11 – 21 years). The most common curve pattern at the time of surgery was structural main thoracic curve (23 patients). Double structural curve and structural thoraco-lumbar/lumbar curves comprised of nine and three patients respectively. The mean preoperative curve magnitude at the time of surgery was 60° (range: 40° – 90°)

### Perioperative parameters

The results of all perioperative parameters is summarised comprehensively in Table 1. The average follow- up was 2.94 years (range: 2–4y). The mean duration of surgery was 148 min (range: 98–175 min). The VAS score was reduced from 1.71 (range: 0–8) to 0.26 at 6 months post-op (range: 0–2). The mean no. of instrumented segments was 9.4 (range: 4–13) and implant density index (IDI) averaged 1.23 (range: 1.15 – 1.50). The harvest from cell saver on recycling yielded an average of 95mls (range: 65–430mls). None of the 35 patients needed any postoperative blood transfusions. Three different instrumentation systems were used in the study population were: Expedium (1), Synergy (11) and K2M (23). The mean amount of ACTIFUSE™ used was 32mls (range: 20–60mls)

### Radiographic parameters

**Fig. 2:**
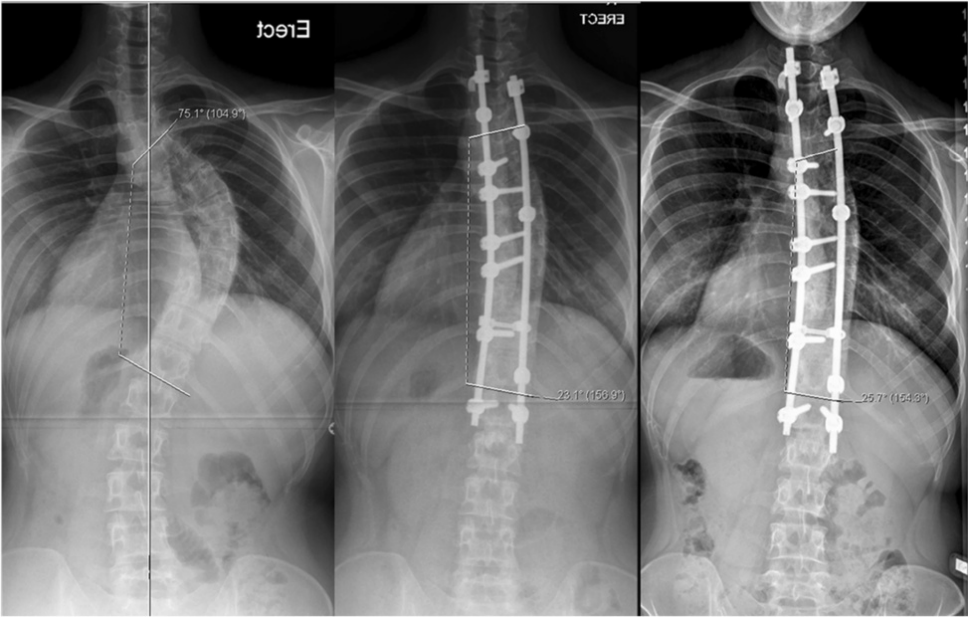
Structural thoracic AIS (*MHHN curve type I*) in 16y/♀ treated with PSF with 66 % correction showing pre-op, immediate post-op and 4 years post-op radiographs

**Fig. 3:**
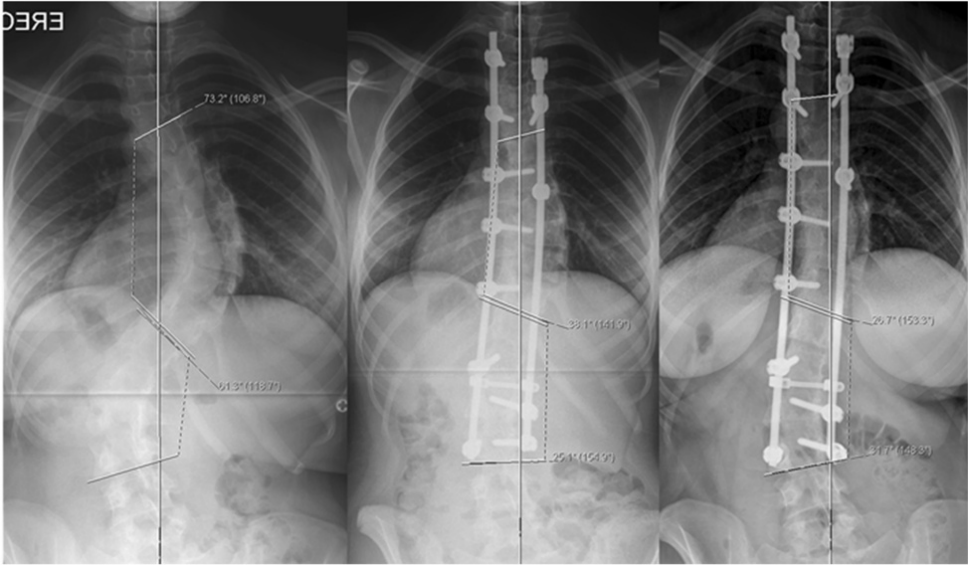
Double structural AIS (*MHHN curve type II*) in 15y/♀ treated by PSF of both curves. 66 % and 50 % correction of main thoracic and compensatory lumbar curves was achieved. The pre-op, immediate post-op and 2.5 years post-op radiographs are shown

**Fig. 4:**
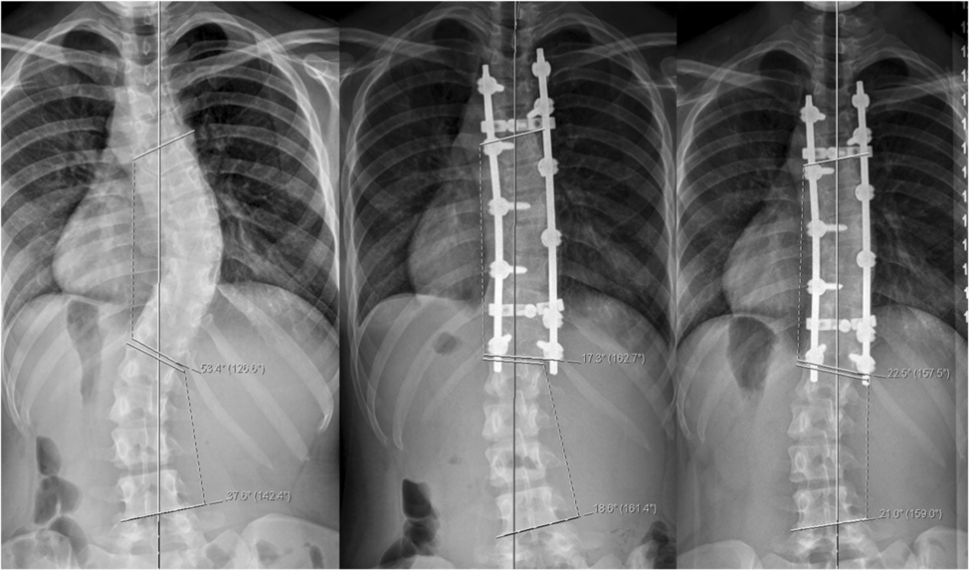
Double structural AIS (*MHHN curve type II*) in 14.5y/♀ treated with *selective thoracic fusion* (65 % correction) with f/u of 3.5 years showing pre-op, immediate post-op and 3.5 years follow-up radiographs

The immediate postop Cobb angle improved to 23.2° (range: 9° – 55°) and new bone formation was seen on plain x-rays as early as 6 weeks in few patients. New bone formation was seen in all cases by 3 months postop follow-up radiographs. Loss of curve correction was seen in 19 patients and remaining 16 had improvement in final Cobb angle with time. The mean loss of Cobb angle at end of study was 2.7° (range: 1°–5°). No patient in the study group had loss of Cobb angle correction by more than 10°. The radiographic parameters in all 35 patients are summarized in Table 2. Case examples of senior author’s algorithm with three curve types (I - III) showing preoperative, immediate postoperative and final follow-up x-rays of AIS treated by PSF and use of low IDI construct (Figs 2, 3 and 4). Instrumentation system had no effect on fusion rates though the numbers were small for the three spinal implant systems used.

**Table 2:** Results - Summary of *radiographic parameters* of all 35 patients

Patient ID	Levels fused	AIS Curve Type MHHN Algorithm	Pre-op Cobb ^le	3 months f/u Cobb ^le	Final F/U Cobb ^le	Total loss of Cobb ^le	Complication	Radiographic fusion	Instrumentation
1001	T2-L1	Type I	75	22	25	3	None	Good	Synergy
1002	T3-L3	Type II	73 & 61	38 & 25	26 & 31	−12	None	Good	Synergy
1003	T2-L2	Type I	56	27	20	−7	None	Good	K2M
1004	T3-L1	Type I	57	15	13	−2	None	Good	K2M
1005	T4-L2	Type I	78	37	30	−7	None	Good	Synergy
1006	T3-L1	Type I	56	22	20	−2	None	Good	K2M
1007	T3-L3	Type I	60	20	22	3	MRSA deep infection	Good despite deep infection	Synergy
1008	T3-L3	Type I	90	12	16	4	None	Good	Synergy
1009	T3-T11	Type II	80 & 55	35 & 38	40 & 21	5	None	Good	Synergy
1010	T3-T11	Type I	74	28	25	−3	None	Good	Synergy
1011	T4-T10	Type II	54 & 45	26 & 28	27 & 25	1	None	Good	Synergy
1012	T2-T12	Type I	64	18	16	−2	None	Good	K2M
1013	T3-L3	Type I	56	19	20	1	None	Good	K2M
1014	T2-T12	Type II	86 & 56	34 & 26	38 & 42	4	None	Good	K2M
1015	T3-L1	Type I	60	26	18	−8	None	Good	K2M
1016	T2-L1	Type I	60	22	20	−2	None	Good	Synergy
1017	T3-T11	Type II	53 & 38	34 & 37	29 & 31	−5	None	Good	K2M
1018	T4-T12	Type II	47 & 38	16 & 26	20 & 20	4	None	Good	Synergy
1019	T2-L3	Type II	68 & 65	32 & 35	30 & 32	2	Right rod broken at T9	Good	K2M
1020	T2-L2	Type I	57	20	16	−4	None	Good	K2M
1021	T4-L2	Type I	64	32	25	−7	None	Good	Synergy
1022	T4-L2	Type I	58	20	22	2	None	Good	K2M
1023	T3-T11	Type II	46 & 36	21 & 21	21 & 21	0	None	Good	K2M
1024	T10-L3	Type III	40	9	10	1	None	Good	K2M
1025	T2-L2	Type I	55	25	28	3	None	Good	K2M
1026	T2-T12	Type II	53 & 37	17 & 18	22 & 21	5	None	Good	K2M
1027	T3-T12	Type I	45	2	4	2	None	Good	K2M
1028	T10-S1	Type III	40	15	18	3	None	Good	Expedium
1029	T3-L3	Type I	60	16	18	2	None	Good	K2M
1030	T4-L1	Type I	55	20	22	2	Unhappy with pain relief	Good	K2M
1031	T3-T11	Type I	58	20	16	−4	Had low back pain	Good	K2M
1032	T11-L3	Type III	50	20	18	−2	None	Good	K2M
1033	T4-T11	Type I	50	18	15	−3	None	Good	K2M
1034	T4-T10	Type I	55	20	22	2	None	Good	K2M
1035	T4-T10	Type I	70	55	52	2	None	Good	K2M

### Complications

**Fig. 5:**
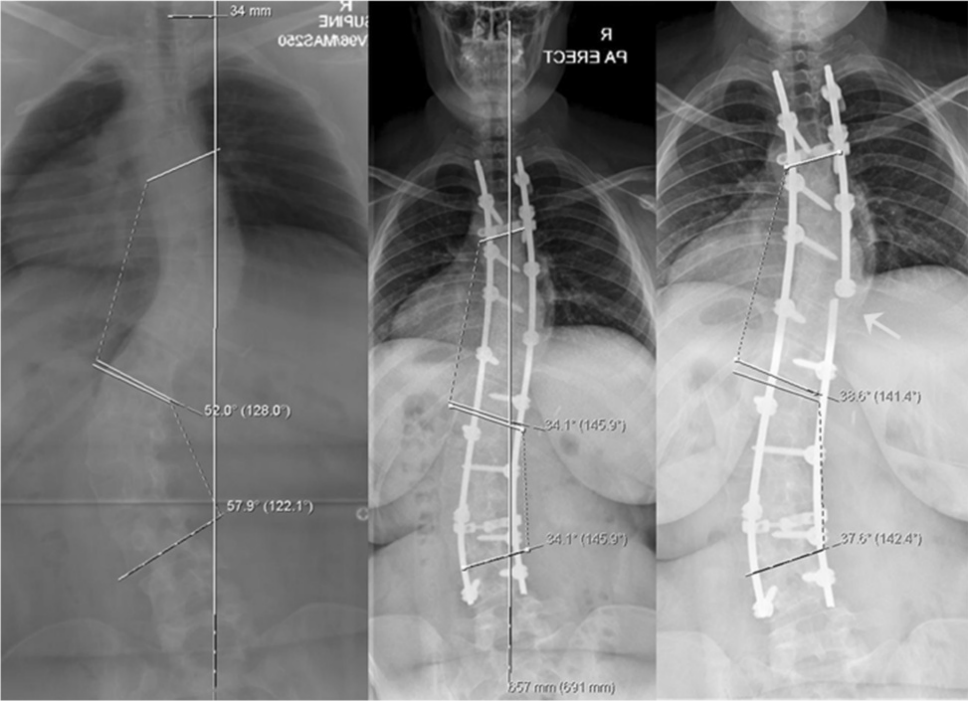
Adverse event – Radiographs of AIS with right rod breakage at 25 months following T3 – L4 PSF
- Late deep MRSA infection warranting removal of all instrumentation at 47 months post-op in a 15 year old male. Sound arthrodesis was seen at the time of implant removal and deep cultures grew MRSA (Fig 6).

There were two adverse events in our case series:

- Implant failure - Without any evidence of pseudarthrosis in a 16 year old female. She presented with a subcutaneously palpable implant and mechanical pain at 25 months postoperatively following a T3 – L4 PSF. Sound arthrodesis with good fusion mass was seen on CT and at revision surgery for exchange of broken rod The relevant preoperative, postoperative radiographs and at the time of revision surgery for right sided rod breakage at 26 months from index surgery are illustrated in Fig 5.

**Fig. 6:**
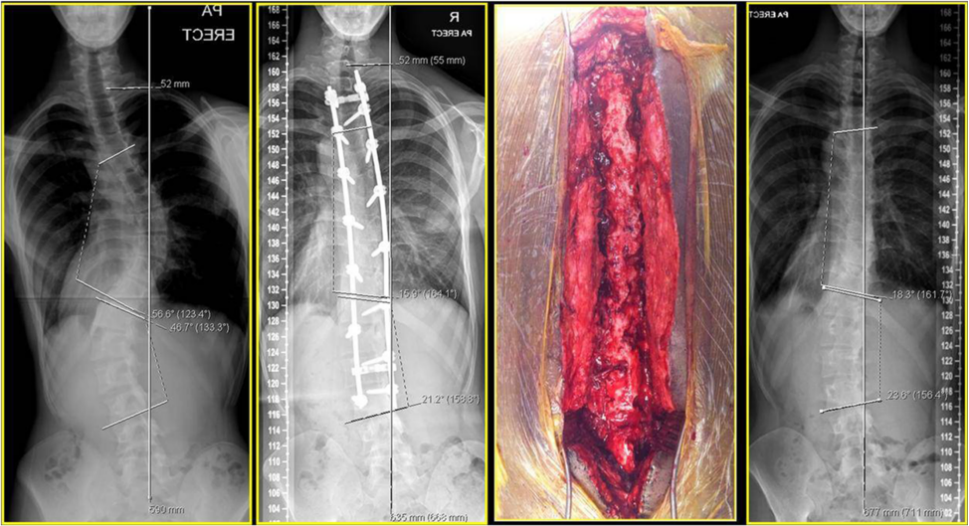
Late deep MRSA infection in a 15/♂ showing pre-op and 47 months post-op x-rays (i.e., immediately prior to implant removal). Solid arthrodesis was seen at implant removal and final x-rays 6 months post implant removal are depicted

## Discussion

We undertook a prospective clinical study to evaluate the therapeutic efficacy of a novel synthetic osteoconductive bone graft expander (SiCaP) for achieving arthrodesis in AIS surgeries. In this group of 35 patients, we did not observe any inflammatory response or any other adverse effects in both genders. We observed osteointegration of SiCaP with native host bone as early as 6 weeks and some evidence of radiographic fusion was seen in all patients by 3 months post-op. The ACTIFUSE™ granules remained circumscribable on plain x-rays at 9 – 12 months and were fully integrated with host bone as solid fusion mass by 24 months.

Radiographic assessment of spinal fusion by plain x-rays is fraught with fallacies and may be inaccurate as they more often tend to over-estimate solid fusion (i.e., false negative for non-unions) [13]. Despite advances in imaging quality, radiographs at best are only two dimensional and dynamic radiographs though helpful in ruling out instability have their own limitations. The main shortcoming of dynamic x-rays includes measurement reliability and disagreement between observers on permissible motion [14]. Computed tomography (CT) is more sensitive in detecting non-union and is investigation of choice to confirm or rule out pseudarthrosis [15]. Three dimensional CT reconstructions are helpful in planning revision surgeries to address symptomatic pseudarthrosis. However enormous radiation exposure precludes its routine use in asymptomatic patients and is reserved for patients with persistent axial back pain and instrumentation failure. A single lumbar spine CT has the irradiation dose equivalent to 240 chest radiographs [16]. Though Magnetic resonance imaging (MRI) is appealing owing to lack of irradiation induced risks, its utility in assessing spinal fusion is inferior to CT scans [17]. Further research is needed to define the MR sequences and magnet strength required to assess spinal fusion in presence of instrumentation and its artifacts.

Lerner et al. compared the osteointegration properties of beta-tricalcium phosphate (b-TCP) and silicated calcium phosphate (SiCaP) as bone graft substitutes in PSF for AIS. Unlike SiCaP, the b-TCP granules were observed to dissolve rapidly (at 6 months) and underwent faster degradation [18, 19]. Similar observations were made by Muschik who reported b-TCP granules to be invisible on radiographs at 8 ± 2 months postoperatively. This faster degradation of b-TCP produced inflammatory response in an animal model [20]. Such an inflammatory response would be detrimental for sound fusion as it can trigger bone resorption. The end volume and quality of such newly formed bone may have poor trabecular architecture and susceptible for breaks/fracture. The silicate substitution at 0.8 % in ACTIFUSE™ was observed to promote accelerated neovascularisation and bone apposition with formation of normal trabecular architecture [11, 19]. It also significantly promoted bioactivity and facilitated adaptive remodelling with better bony in-growths in comparison to hydroxyapatite (HA). We mixed SiCaP and locally harvested bone in 60:40 ratio prior to laying over the instrumented area. Posterior decortication of laminae and transverse processes exposes the bleeding cancellous bone creating an ideal environment for new bone formation facilitating linkage between native bone and bone graft substitute. Korovessis observed that HA in combination with local bone and bone marrow aspirates (BMA) did not produce satisfactory arthrodesis in postero-lateral fusion unlike SiCaP which produced consistent results and good fusion mass in similar surgeries [21].

Lerner et al. used 20 – 40mls of SiCaP mixed with locally harvested bone plus BMA from vertebral bodies in 21 patients who underwent posterior spinal fusion for AIS [19]. BMA possess osteoinductive properties and the true role of SiCaP in causing arthrodesis when mixed with such an osteogenic agent cannot be determined definitively/is questionable [22]. We did not use BMA in any of our 35 patients in this series and all but one had good/excellent arthrodesis with similar quantities of SiCaP (average 32mls). This conclusively establishes that solid fusion does happen with isolated use of SiCaP when mixed with locally harvested bone in scoliosis surgeries.

Finally, the gold standard iliac crest bone graft (ICBG) was associated with significant donor site morbidity in adolescents that limited activities of daily living (ADL) in at least 21 out of 87 patients observed by Skaggs [9]. The mean VAS was 4 and up to 10 % were on regular analgesics even at 4 years post-op from the time of index surgery. They concluded that the true dimensions of pain and suffering from other complications associated with ICBG was significantly under-reported in published literature. All patients in our series were pain-free except two and had stopped all analgesics by 6 months post-op. Only 14 patients reported mild pain/discomfort at 6 months and the mean VAS score at final follow-up was 0.23 (range: 0 – 2). Only two patients had moderate pain that warranted intake of over the counter NSAIDs.

Implant density index refers to the number of fixation anchors used to correct the scoliotic deformity. It is defined as the ratio of number of fixation anchors to the number of vertebral segments fused [23]. Larger the IDI, better is the degree of Cobb angle correction and lesser the loss of correction with time owing to superior fixation and enhanced pull-out strength imparted by the anchors. Constructs with IDI of <1.5 are considered to be of lower implant density and many studies have questioned the need for bilateral screws in every vertebra for AIS correction [24, 25]. There was negligible loss of Cobb angle correction at final follow-up and was within the measurement error (i.e., mean loss of correction was 2° [range: 1°–5°]) despite low IDI construct. One patient with instrumentation failure had exploration of fusion mass and exchange of broken rod for a prominent implant was found to have sound fusion at the time of revision surgery (Fig 6).

The use of low IDI constructs yielded significant cost-savings without any compromise in our clinical results. The market price of 20mls of ACTIFUSE™ is equivalent to that of one pedicle screw. Utilizing the synthetic bone graft substitute for the entire study cohort amounted to the price of one and half pedicle screws (i.e., 30mls of ACTIFUSE™ = 1.5 pedicle screw in terms of cost) which was more cost-effective in-comparison to a scenario wherein one were to have used bilateral anchors at each vertebral segment.

Our study was not without limitations. Firstly we did not peform any *power analysis* to determine the number of AIS cases treated by PSF that would be needed to detect one pseudoarthrosis. Secondly we did not perform CT scans in all patients to assess fusion and the role of CT was confined only for evaluation of symptomatic patients who had either instrumentation failure or persistent pain. Ethical considerations and hazards of irradiation of asymptomatic patients esp. in reproducible age group was the main hurdle to the same. We would not be surprised if any REC would approve such a study. Thirdly two of our patients with persistent pain used NSAIDs owing to intolerance to opioid based medications (severe itching in one and drowsiness in the other). NSAIDs consumption and nicotine comsumption would interfere would fusion rates (esp. in early phase of healing) from many well documented clinical studies [26, 27]. Consumption of nicotine (smoking/chewing) status was not collected in any of our patients owing to the reluctance of majority of teenagers to be upfront and disclose inviting wrath from their parents/care-givers. And finally we did not have a control group to evaluate the results of SiCaP used PSF vs. controls who underwent PSF. The control group would ideally be a age, curve severity and sex matched AIS patients were operated by PSF with use of ICBG. Performing such a prospective comparative study (i.e., at least a LoE II if not a randomized controlled study) is desired to truly determine the role of SiCaP in spinal deformity surgeries. This would undoubtedly be grounds for further research and this pilot study is a first step towards that goal having established the safety profile of SiCaP without any untoward adverse events with its clinical use.

## Conclusion

In conclusion, SiCaP’s use was safe and it produced a predictable sound arthrodesis without any adverse effects or inflammatory response in all 35 patients in our series treated by PSF. The surgical results were equivalent or superior and consistent with published historical studies associated with use of gold standard ICBG. Use of SiCaP had distinct advantages in minimising complications reported with ICBG harvest. Use of low IDI instrumentation constructs was not associated with unacceptable (i.e., >10°) loss of correction or increase in pseudarthrosis rates at 2 years. There was substantial cost savings without any compromise in clinical outcomes.
